# Evaluation of extended-spectrum *β*-lactamase producing bacteria in feces of shelter dogs as a biomarker for altered gut microbial taxa and functional profiles

**DOI:** 10.3389/fmicb.2025.1556442

**Published:** 2025-03-24

**Authors:** Reta Abdi, Srinka Datta, Akshaykumar Zawar, Pratap Kafle

**Affiliations:** ^1^Biomedical Sciences College of Veterinary Medicine, Long Island University, Brookville, NY, United States; ^2^GeneSpectrum Life Sciences LLP, Pune, Maharashtra, India; ^3^Shreiber School of Veterinary Medicine, Rowan University, Mullica Hill, NJ, United States

**Keywords:** 16S amplicon sequencing, ESBL bacteria, gut microbiota, alpha diversity, microbiome function, shelter dogs

## Abstract

**Background:**

The USA is home to 83–88 million dogs, with 3–7 million living in shelters. Shelter dogs move through the supply chain from their geographical origin to adoptive homes, with possible exposure to pathogens and shift in their gut microbiota. However, research in this area is limited. This study examined the effects of intestinal colonization by ESBL bacteria on gut taxa abundance, diversity, and functions in 52 shelter dogs of various ages, sexes, and fertility statuses.

**Methodology:**

We isolated fecal DNA, sequenced their 16S, processed the sequences using DADA2, identified taxa profiles in each dog by Phyloseq, and analyzed Chao1, Shannon, and Simpson alpha diversity by ggplot2 and Wilcoxon test. We analyzed beta diversity using Bray–Curtis dissimilarity matrix from the vegan package. Differential abundance of taxa, gut microbiome functions, and differential abundance of microbiome functions were analyzed using DESeq2, PICRUSt2, and ALDEx2, respectively, with Wilcoxon rank and Kruskal-Wallis tests for comparisons between dog groups.

**Results:**

Firmicutes (69.3%), Bacteroidota (13.5%), Actinobacteriota (6.77%), Proteobacteria (5.54%), and Fusobacteriota (4.75%) were the major phyla in the gut of shelter dogs. ESBL bacteria colonized dogs had reduced gut microbiota alpha diversity than non-colonized dogs. The abundance levels of the following phyla (Proteobacteria, Deferribacterota, Bacteroidota, Fusobacteriota, and Spirochaetota), class (Gammaproteobacteria, Bacteroidia, Deferribacteres, Brachyspirae, and Fusobacteria), and families (*Enterobacteriaceae*, *Peptostreptococcaceae*, *Lactobacillaceae*, *Lachnospiraceae*, *Prevotellaceae*, and *Peptostreptococcaceae*) were significantly (*p* < 0.05) varied between the two dog groups. Further stratified analysis by age, sex, and spaying/neutering status influenced the abundance of taxa in ESBL bacteria colonized dogs, indicating these covariates act as effect modifiers. Most gut metabolic and biosynthetic pathways were downregulated in ESBL bacteria colonized dogs compared to non-colonized dogs. However, alpha-linolenic acid metabolism and shigellosis, fluorobenzoate degradation, allantoin degradation, toluene degradation, glycol degradation, fatty acid and beta-oxidation, and glyoxylate metabolism bypass pathways were increased in dogs colonized by ESBL bacteria.

**Conclusion:**

Colonization by ESBL bacteria marks altered gut microbiota. Dog’s demography and fertility status modify the alterations, indicating host factors and ESBL bacteria interplay to shape gut microbiota. ESBL bacteria or other factors reprogram gut microbiome functions through down and upregulating multiple metabolic and biosynthesis pathways to promote ESBL bacteria colonization.

## Introduction

The USA is home to 83–88 million dogs,^1^ with 3–7 million residing in shelters (Rowan and Kartal, 2018; Rodriguez et al., 2022). Many shelter dogs come from diverse geography (Rowan and Kartal, 2018), encountering fluctuating care (Pesavento and Murphy, 2014; Rowan and Kartal, 2018; Lamon et al., 2021), stress (Pesavento and Murphy, 2014; Protopopova, 2016; Lamon et al., 2021), diet, and ill health/diseases (Pesavento and Murphy, 2014; Lamon et al., 2021) as they travel in supply chains from their origin to adoptive homes. As a result, chronically stressed dogs with history of predator, ill health, and antimicrobial overuse enter shelters in the U.S. (Pesavento and Murphy, 2014; Lamon et al., 2021). Entry of stressed dogs into shelters suggests that antimicrobial overuse/misuse may occur as a preventive measure to control pathogen flare-ups in shelters (Pesavento and Murphy, 2014). In general, antimicrobial overuse promotes antimicrobial resistant (AMR) bacteria and AMR genes (Yu et al., 2014; Wuethrich et al., 2021) and misuse of antimicrobials and non-antimicrobial drugs in dogs has been already documented in the USA (Sykes, 2013; Lavigne et al., 2021). One of the most concerning AMR bacteria is ESBL-producing bacteria, which are widespread in the global ecosystem and pose a threat to both humans and animals (Podolsky, 2018; Larsson and Flach, 2022). The odds of human mortality by ESBL producer bacteria is higher (odds ratio = 1.70) than non-ESBL producer bacteria (Ling et al., 2021). The global prevalence of ESBL producing bacteria in dogs is rising, posing a threat to both animal and human health due to the potential zoonotic bacteria transmission from dogs to their owners (Salgado-Caxito et al., 2021).

As shelter dogs encounter diverse geographies, pathogens, and substandard conditions during their transit, they have higher chances of acquiring ESBL bacteria, as ESBL bacteria are already widespread in the ecosystem (Podolsky, 2018; Larsson and Flach, 2022). Shelters, as key part of the shelter dogs supply chain, could be high-risk environments for the transmission of ESBL-producing bacteria since stressed dogs from many geographical sources are brought together in shelters (Freestone et al., 2008; Verbrugghe et al., 2012; Pesavento and Murphy, 2014; Protopopova, 2016; Jašarević et al., 2017; Molina-Torres et al., 2019; Sarkodie et al., 2019; Lamon et al., 2021).

Furthermore, gut is the epicenter of AMR due to the horizontal transfer of AMR genes among the trillion gut microbiota in the gut (Carlet, 2012). Shelter dogs can share their gut bacteria during mingling to each other and they can also share such gut microbiota ultimately with their owners (Jiang et al., 2022) including pathogenic ones (Jacob and Lorber, 2015). Generally, dogs harbor about 10^12^–10^14^ microbes in number in their gut microbiota (Suchodolski, 2011; Garrigues et al., 2022), comprising of 1,000 species, and encoding about 3 million genes that play many roles for the host (Rowland et al., 2018; Chen et al., 2024). They produce numerous enzymes and metabolites that the host cannot synthesize, supporting neural, humoral, immunological functions of the host, as well as defense against invading pathogen through competition or direct killing (Tremaroli and Bäckhed, 2012; Pickard et al., 2017; Afzaal et al., 2022). They also synthesize vitamins, enhance metabolic pathways of amino acid, fatty acid, and breakdown of polysaccharides and polyphenols for energy extraction from the food, and among other processes (Tremaroli and Bäckhed, 2012; Pickard et al., 2017; Rowland et al., 2018; Afzaal et al., 2022; Chen et al., 2024). Overall, the gut microbiota supports the development, morphogenesis, physiology, metabolism, and homeostasis of various organs and systems, including the brain, glands, hormones, heart, lung, liver, pancreas, bones, muscles, kidney, bladder, and reproductive organs (Sommer and Bäckhed, 2013; Afzaal et al., 2022). Unfortunately, multiple factors including stress (Freestone et al., 2008; Verbrugghe et al., 2012; Jašarević et al., 2017; Molina-Torres et al., 2019; Sarkodie et al., 2019), antimicrobial use (Yu et al., 2014; Wuethrich et al., 2021), and invasion by pathogens (Pickard et al., 2017) can disrupt the gut microbiota abundance, composition, and their metabolic and biosynthesis pathways, but research in this area is limited in shelter dogs. The specific determinants and mechanisms of fluctuations in relative abundance of gut microbiota and their functional consequences are not known in shelter dogs. Overall, shelter dogs are valuable in biomedical research as they offer insights into how life experiences during transit (both positive and negative), mingling, and fluctuations in pathogen colonization and AMR genes levels influence their microbiome, overall health, and public health. However, there is limited information on the dynamics of intestinal colonization by ESBL-producing bacteria in shelter dogs, their impact on gut microbiota, and on overall health. This gap underscores the need for further studies to guide shelter improved management practices and control the risk of AMR bacteria transmission. This study examined the effects of intestinal colonization by ESBL bacteria on gut taxa abundance, composition, and microbiome functions in 52 shelter dogs of various ages, sexes, and fertility statuses in Long Island, New York.

## Methodology

### Shelter dogs sampling

This cross-sectional study was conducted at shelter dogs in Long Island New York, USA. After initial dog demographic assessment, inclusion criteria were established to ensure sample homogeneity and statistical validity. The study sample population comprised of 52 (*n* = 54) clinically healthy dogs sampled randomly from a volunteer shelter, stratified by age, sex, fertility status and ESBL bacterial colonization status. Accordingly, we sampled 27 females (8 intact, 19 spayed), 25 males (8 intact, 17 neutered), ESBL positive (*n* = 12) and ESBL negative dogs (*n* = 40), and with age ranging from 1 to 60 months. Age stratification was performed as follows: 1–3 months (*n* = 17), 4–6 months (*n* = 16), 7–12 months (*n* = 8), and 24–60 months (*n* = 11). Fecal samples (~1 g) were collected directly from rectum of 52 dogs using sterile techniques, following protocols approved by the Institutional Animal Care and Use Committee (IACUC ID#19-15). We swab-plated the feces sample of each dog on CHROMagar ESBL media to categorize dogs into ESBL positive and ESBL negative since this bacteriological medium reliably detects ESBL bacteria carrier individuals in their gut (Mannathoko et al., 2023).

### DNA extraction, PCR amplification, and 16S rRNA gene sequencing

Of 1 g feces, ~200 mg was used for genomic DNA isolation using ZymoBIOMICS DNA Kits following the manufacturer’s standardized protocol. We amplified the V3–V4 hypervariable regions of the 16S rRNA genes using universal primers 341F (5’-CCTACGGGNGGCWGCAG-3′) and 805R (5′- GGACTACHVGGGTWTCTAAT −3′), generating 465 bp amplicons. PCR ingredients and PCR cycling conditions were optimized to minimize bias and ensure consistent amplification across all samples as described previously (Hugerth Luisa et al., 2014). Subsequently, 25 μL of reaction mixture of each sample (2.5 μL of each primer, 12.5 μL of PCR Premix, 25 ng of template DNA and ddH_2_O [for volume adjustment]) was amplified by PCR targeting 16S gene at an initial denaturation at 98°C for 30 s; followed by 32 cycles of denaturation at 98°C for 10 s, annealing at 54°C for 30 s and extension at 72°C for 45 s; with a final extension for 10 min at 72. The PCR products of each sample were evaluated using 2% agarose gel electrophoresis. AMPure XT beads (Beckman Coulter Genomics, Danvers, MA, United States) were used for purifying the PCR products from primers, primer dimers, dNTPs, small and large DNA fragments, and other contaminants. Qubit (Invitrogen, Carlsbad, CA, United States) was used for quantifying the purified 16S PCR products. Then Illumina-specific adapters and sample-specific barcodes (indices) were used to amplify the purified 16S by second round PCR step followed by purifying the indexed amplicon products again using AMPure XT beads and verified the amplicon size using Agilent 2,100 Bioanalyzer (Agilent, Palo Alto, CA, United States). Subsequently, individual libraries were normalized based on concentration and pooled them into a single sequencing library. Quantity of the pooled amplicon library were quantified using the library quantification kit for Illumina (Kapa Biosciences, Woburn, MA, United States). After amplifying the library and denaturing and diluting the final library to the appropriate concentration, they were loaded onto an Illumina NovaSeq platform for high-throughput sequencing using 2 × 250 bp paired end reads mode (LC Sciences, Houston, Texas). Quality control measures included internal standards and negative controls to ensure sequencing accuracy and detect potential contamination.

### Bioinformatics analysis pipeline

#### FASTQ file

Raw 16S sequence data from the Illumina Novaseq FASTQ file underwent rigorous assessment for quality using FastQC v0.11,^2^ with specific attention to sequence length, poor sequence reads (quality score < 20), and ambiguous/undefined base insertion (Vigil et al., 2024).

#### DADA2 for processing raw data of 16S sequences

To further improve the FASTQ reads quality, we utilized DADA2 (version 1.33.0) (Callahan et al., 2016) for comprehensive sequence processing. DADA filtered and trimmed poor quality sequences, truncated forward and reverse reads to 250 bp, removed sequencing errors and chimeric sequences, dereplicated the sequences, and merged pair end reads to maintain sequence quality consistency. Finally, we constructed amplicon sequence variant (ASV) table in rows along with each dog’s demographic factors in columns.

#### Taxonomic classification of gut microbiota in reference to the SILVA database

We aligned our processed 16S sequences (ASVs) of each shelter dogs against the SILVA reference database (Release 138)^3^ using a Naive Bayes classifier. This alignment helped for the classification of gut microbiota taxa at various taxonomic level (phylum, class, order, family, and genus), depending on the resolution provided by the sequence data identity and the reference database (Yarza et al., 2014; Glöckner et al., 2017).

### Data integration and statistical analysis

#### Phyloseq for integrating comparison groups of shelter dogs into 16S ASV table

We created a comprehensive dataset that supports various statistical analyses by phyloseq (version 1.48.0), by inserting the taxonomic classifications and sample metadata (dog demography, risk factors or comparison groups) into the ASV table for downstream data analysis (McMurdie and Holmes, 2013). Quality filtering included removal of dog samples with fewer than 30,000 sequence reads from the analysis to ensure adequate sequencing depth and elimination of taxa with zero counts to reduce noise and improve statistical power (Manor et al., 2020). Since the sequence depth of each shelter dogs sample varied, we rarefied our phyloseq data to an even depth across all samples. Rarefaction standardizes the number of sequences per sample, which is essential especially in studies where sequencing depth varies (Willis, 2019). Data normalization using rarefaction ensured comparable sequencing depth across all samples for fair comparisons of diversity across all samples.

### PICRUSt2 for mining/predicting gut microbiome functions in KEGG and MetaCyc database

The functions and pathways present in the gut microbiome of shelter dogs were predicted using PICRUSt2 and its enhanced version ggpicrust2 (version 1.7.3) by modeling the 16S sequence data of each dog sample (Langille et al., 2013; Yang C. et al., 2023), in reference to KEGG database^4^ and MetaCyc database^5^ (Altman et al., 2013).

### R package for alpha diversity, beta diversity, and microbiome functions analysis

To compare the unique and shared ASVs among dog groups (age, sex, ESBL, and fertility status), Venn diagrams were generated using the ggvenn_pq function from the MiscMetabar package.

#### Alpha diversity analysis

The phyloseq formats of the 16S sequence data described above were visualized by the plot_richness function different aspects of diversity measures in R packages (McMurdie and Holmes, 2013; Wen et al., 2023). We used ggplot2 for alpha diversity indices analysis (Wilkinson, 2011). Of multiple alpha diversity indicator indices, we used Shannon, Simpson, and Chao1 for evaluating richness and evenness of microbiota taxa across different comparison groups in shelter dogs, such as sex, fertility, age, and ESBL status (Kers and Saccenti, 2022). Comparisons between different dog groups were conducted using the Kruskal-Wallis test, which is widely used for non-parametric data and allows comparison across multiple groups without assuming normality.

#### Beta diversity analysis

For beta diversity index analysis, we first transformed the phyloseq data into relative abundance data since data transformation allows for comparisons by accounting for the relative proportions of different taxa, rather than their absolute counts. Non-metric multidimensional scaling (NMDS) was performed using the Bray–Curtis dissimilarity matrix to visualize dissimilarities/differences in microbial communities across samples (Kers and Saccenti, 2022). Differences in microbial composition between groups were statistically tested using PERMANOVA with 999 permutations using the adonis2 function from the vegan package, which is suitable for multivariate community data and accounts for group-level variation. NMDS ordination plots were generated for different groups using vegan package version 2.6.2^6^ of R, providing a visual representation to compare the similarities and differences in microbial compositions (Dixon, 2003). Ordination plots were generated using ggplot2 (Wilkinson, 2011) with 95% confidence ellipses for each group.

#### Gut microbiota differential abundance analysis by taxa

DESeq2 was used to count and analyze taxonomic differential abundance of 16S sequence data by taxa to identify significantly abundant (or differences in) taxa at the Phylum, Class, and Family levels across the groups (sex, fertility status, age, and ESBL status) (Abegaz et al., 2024; Wirbel et al., 2024). DESeq2 applies a negative binomial model to normalized data, accounting for biological variability in relative abundance measures. To control for false positives, we applied Benjamini-Hochberg (BH) false discovery rate (FDR) correction, which reduces Type I error when performing multiple comparisons.

#### Gut microbiome functions/pathways analysis

We used the improved version PICRUSt2 called ggpicrust2 (version 1.7.3). to test the differences in functional/pathway abundances (i.e., differential abundance) between comparison groups in our dataset using non-parametric statistical tests and visualization of the microbiome functional profiles for each comparison groups in shelter dogs (Yang C. et al., 2023). We utilized a more conservative and precise analysis tool within ggpicrust2 called ANOVA-like differential expression (ALDEx) using Wilcoxon rank and Kruskal Wallis tests for comparing differences in abundance of functions/pathways (Fernandes et al., 2013; Nearing et al., 2022).

## Results

### Initial data processing and filtering

The initial dataset consisted of 5,116,824 raw 16S rRNA gene sequences. After quality analysis and filtering to ensure Phred score > 30, 3,952,710 clean sequence reads were obtained. We performed denoising, merging, removal of chimeric sequences and refined the dataset, retaining 2,117,442 high-quality sequences, representing 41.38% of the original dataset. The high-quality sequences in the dataset were clustered into 6,796 Operational Taxonomic Units (OTUs). A further filtering step was applied to reduce noise from rare OTUs and focus on prevalent taxa, OTUs with fewer than 30,000 counts across all samples were excluded. This filtering resulted in the retention of 6,557 OTUs for downstream analyses (Table 1).

**Table 1:** Counts of initial raw, cleaned, removed, and retained data of 16S sequences.

Step	Counts
Initial dataset received from sequencing machine	5,116,824
Clean sequence reads (Phred > 30)	3,952,710
High-quality sequences retained	2,117,442
Percentage of original dataset	41.38%
OTUs identified	6,796
Filtering threshold (counts)	30,000
OTUs retained after filtering	6,557

### Unique and shared gut microbiota among age, sex, fertility status, or ESBL groups

**Figure 1:**
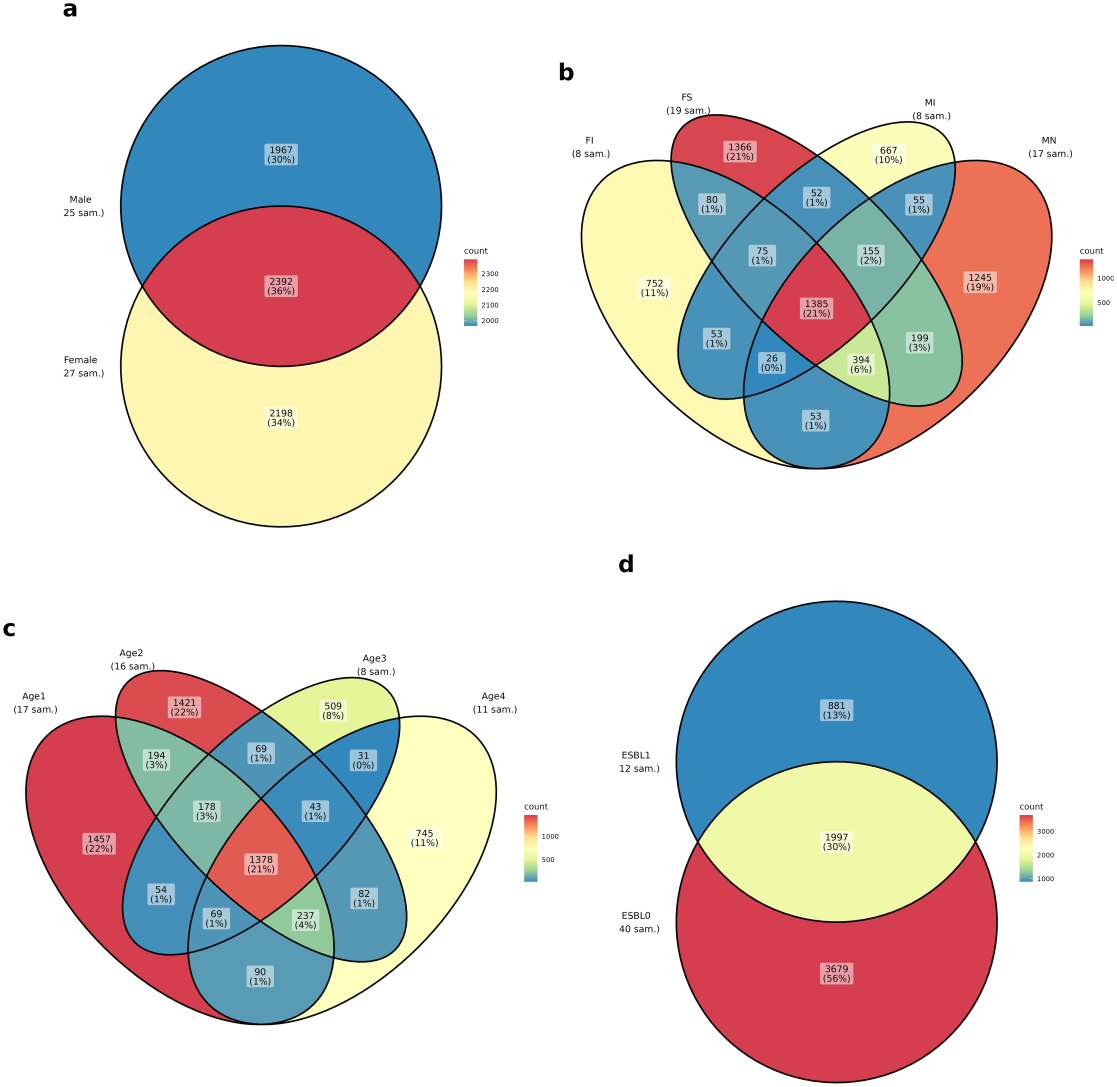
Venn diagram of ASVs shared among the four groups: **(a)** sex, **(b)** fertility status (spayed females = FS, neutered males = MN, intact females = FI, intact males = MI), **(c)** age (1–3 months = age 1, 4–6 months = age 2, 7–12 months = age3, 12–60 months = age 4), and **(d)** ESBL status in shelter dogs. For sample size (*n*) of dogs in each group, look at sampling under the methodology above.

The Venn diagram analysis showed unique and shared ASVs by age, fertility, sex, and ESBL status. Female dogs exhibited a higher number of unique ASVs (2198) than male dogs (1967). Both sexes shared 2,392 ASVs. Spayed females (FS) and neutered males (MN) showed the (1,366 and 1,245, respectively) than intact females (FI) and intact males (MI) (=752 and 667, respectively). A total of 1,385 ASVs were shared among the four fertility groups. Overall, 1,378 ASVs were shared among the four age groups. Puppies under 3 months had the highest ASVs (1,457), followed by 4–6 months of age, 7–12 months of age, and 24–60 months of age (decreasing in that order). ESBL-negative dogs (ESBL0) had the highest number of unique ASVs (3,679) than ESBL-positive dogs (ESBL1 = 881). Dog with and without ESBL shared 1997 ASVs (Figure 1).

### Alpha diversity

**Figure 2:**
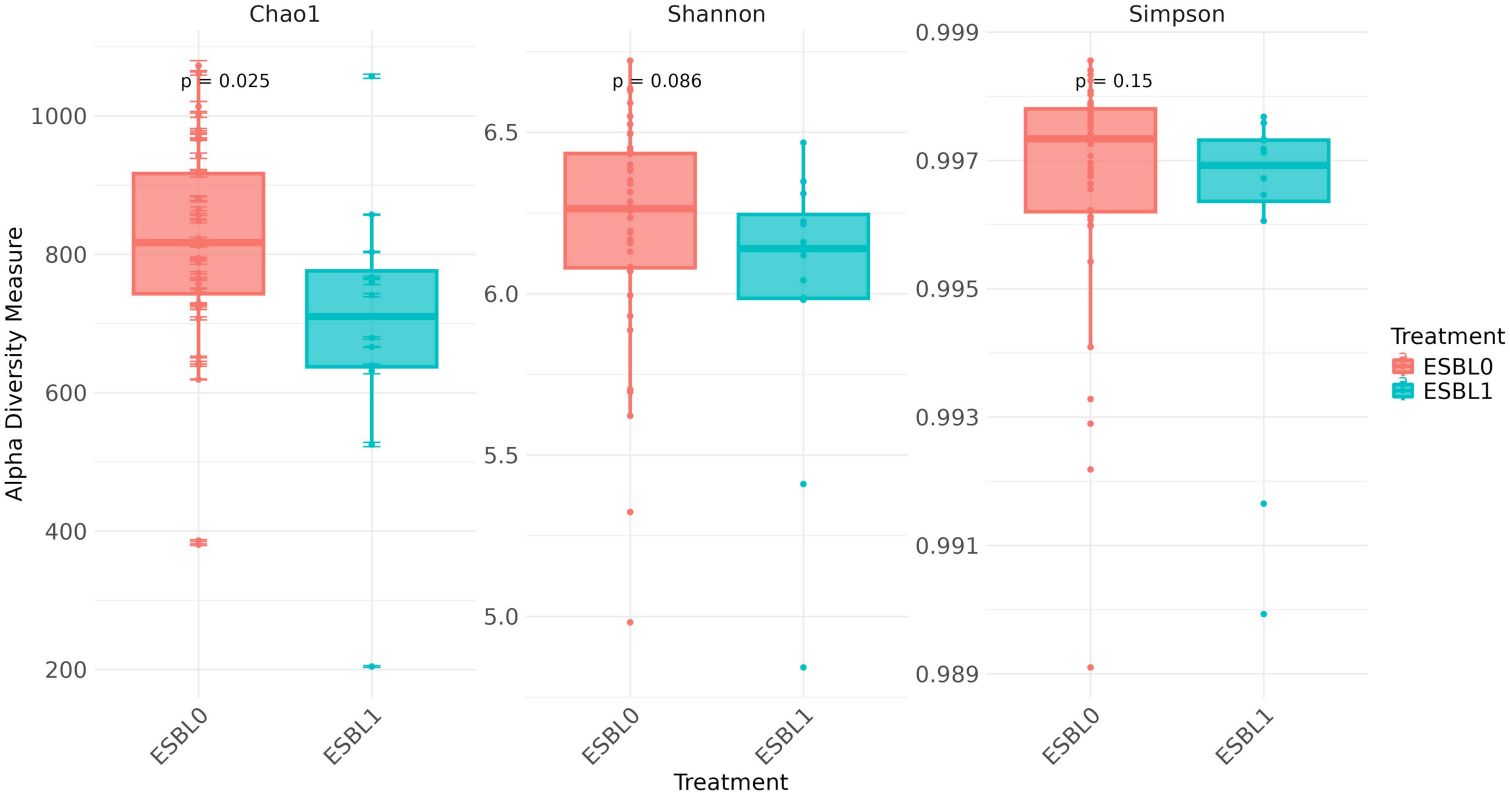
Boxplots of alpha diversity metrics with and without ESBL-bacteria colonization in the gut microbiota in shelter dogs. Boxplots display the Chao1, Shannon, and Simpson indices for ESBL-positive (*n* = 12) and ESBL-negative (*n* = 40) dogs. Statistical significance was determined using the Kruskal-Wallis test. Chao1 index showed a significant reduction in ESBL-positive dogs (*p* =  0.025), while Shannon (*p* = 0.086) and Simpson (*p* = 0.1517) indices showed no significant differences.

Regardless of age, sex, and fertility status, dogs colonized by ESBL-producing bacteria harbored significantly lower bacterial alpha diversity compared to ESBL-negative dogs, as assed by Chao1 index (*p =* 0.025). However, no significant differences were observed using the Shannon index (*p =* 0.086) or Simpson index (*p =* 0.15), indicating that microbial diversity may vary based on ESBL colonization status and the chosen diversity metric (Figure 2).

**Figure 3:**
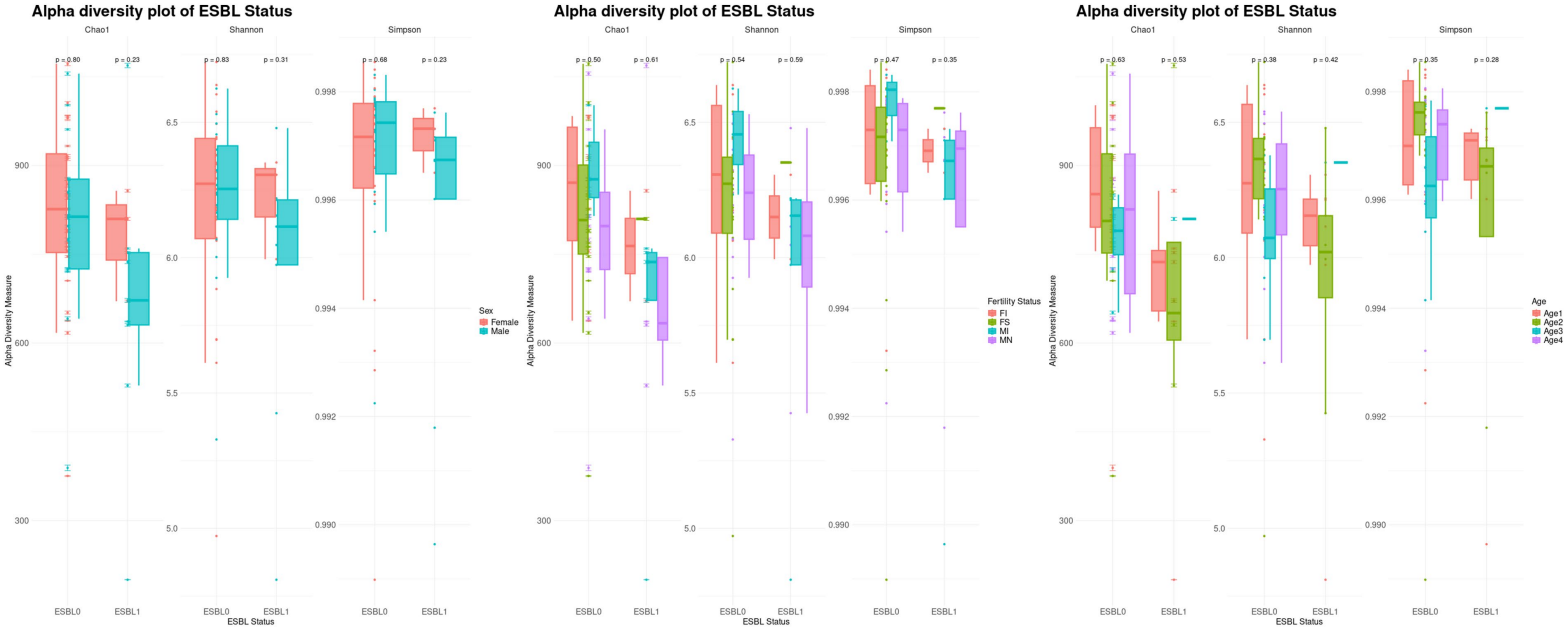
Boxplots of stratified alpha diversity analysis of ESBL-positive and ESBL-negative dogs by sex, fertility, and age. Alpha diversity metrics (Chao1, Shannon, Simpson indices) compared across ESBL-positive and ESBL-negative dogs, further stratified by sex (male vs. female), fertility status (intact vs. spayed/neutered), and age groups. Statistical significance was tested for significance using Kruskal-Wallis test.

Alpha diversity analysis by further stratification of ESBL-positive dogs by sex, fertility, and age showed that female dogs had higher alpha diversity compared to males when colonized by ESBL-producing bacteria. Spayed females colonized by ESBL-producing bacteria had higher diversity than intact females, while intact males colonized by ESBL-producing bacteria had higher diversity than neutered males. Among ESBL-positive dogs, alpha diversity was higher in the 7–12 months age group, followed by those under 3 months, and then 4 to 6 months (decreasing in that order). However, these differences in diversity were not significant according to Chao1, Shannon, and Simpton indices (Figure 3).

### Gut microbiota abundance by phyla, class, and family

Fifteen phyla, 22 classes, 119 bacterial families, and 285 genera were identified in the gut microbiota of shelter dogs. Intestinal colonization by ESBL-producing bacteria was associated with significant changes in microbial dynamics across the phylum, class, and family of gut microbiota, where the extent of variations in microbiota abundance between ESBL-positive and ESBL-negative dogs was also influenced by the sex, age, and fertility status of the dogs.

#### Phyla

**Figure 4:**
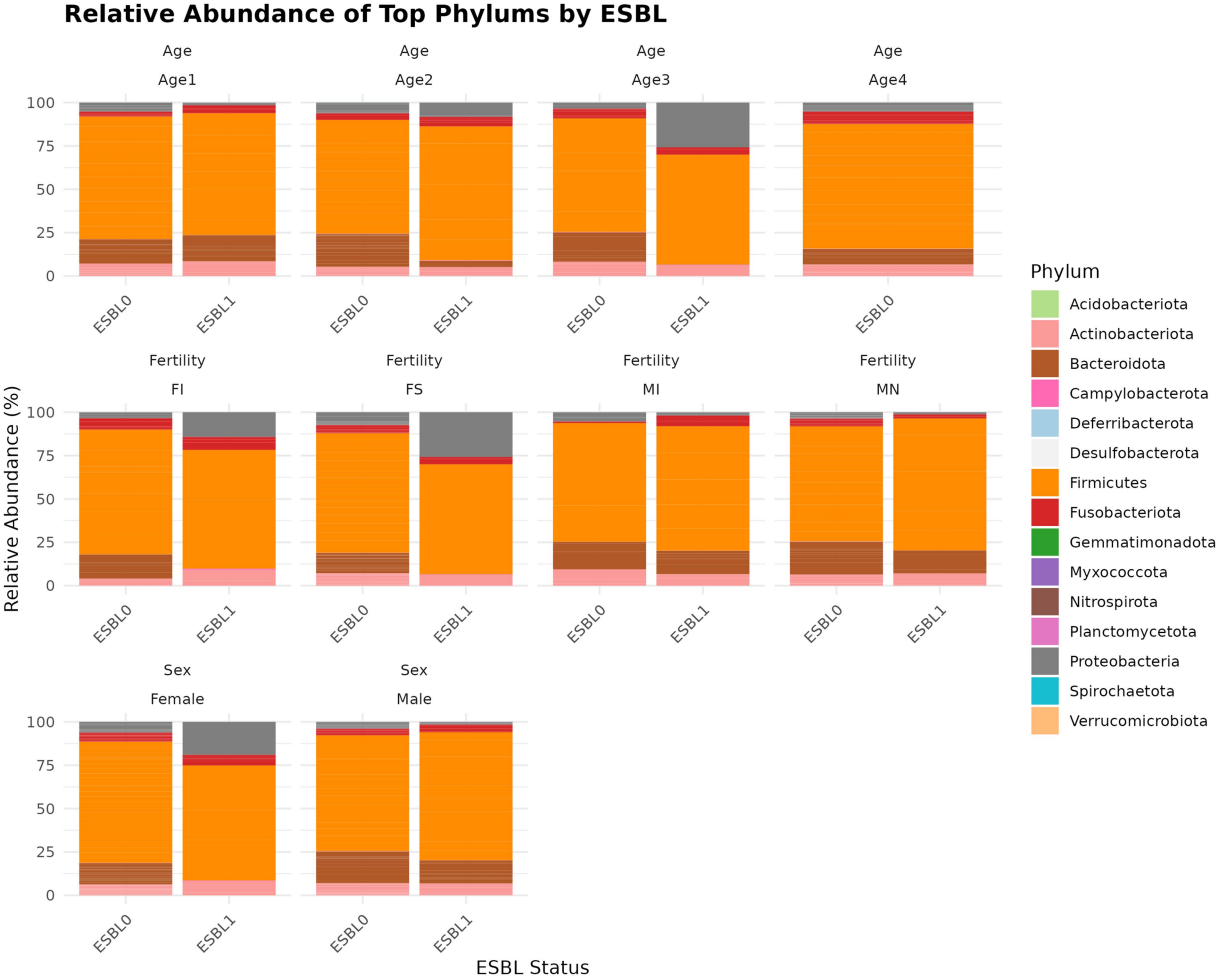
Relative abundance and composition of the gut microbiota altered at phylum level in ESBL-positive and ESBL-negative dogs as stratified by sex, fertility, and age. The relative abundance of major bacterial phyla in ESBL-positive (*n* = 12) and ESBL-negative (*n* = 40) dogs. Statistical significance for group differences was determined using DESeq2 with Benjamini-Hochberg FDR correction (adjusted *p* < 0.05).

A total of 15 phyla were identified (Figure 4), with the five most prevalent being Firmicutes (69.3%) > Bacteroidota (13.5%) > Actinobacteriota (6.77%) > Proteobacteria (5.54%) > Fusobacteriota (4.75%), which together accounted for 99.86% of the gut microbial community in shelter dogs. Gut colonization by ESBL bacteria led to a decrease in Bacteroidota among ESBL-positive dogs (8.01%) compared to ESBL-negative dogs (15.13%). The levels of Proteobacteria phylum rose to 10.85% in ESBL-positive dogs compared to 3.95% in ESBL-negative dogs and decreased to 4.92% in neutered dogs from 7.45% in intact males. Further stratified analysis by sex and age among dogs colonized by ESBL bacteria indicated that Proteobacteria levels significantly increased in females and in dogs aged 7 to 12-months, while Bacteriodata levels decreased across all dogs, regardless of fertility status, sex, or age except in those aged 1 to 3 months. Overall, Proteobacteria (*p =* 0.0002), Deferribacterota (*p =* 0.004), Bacteroidota (*p =* 0.011), Fusobacteriota (*p =* 0.02), and Spirochaetota (*p =* 0. 03) significantly changed in dogs colonized by ESBL bacteria than the non-colonized dogs (Figure 4).

#### Class

**Figure 5:**
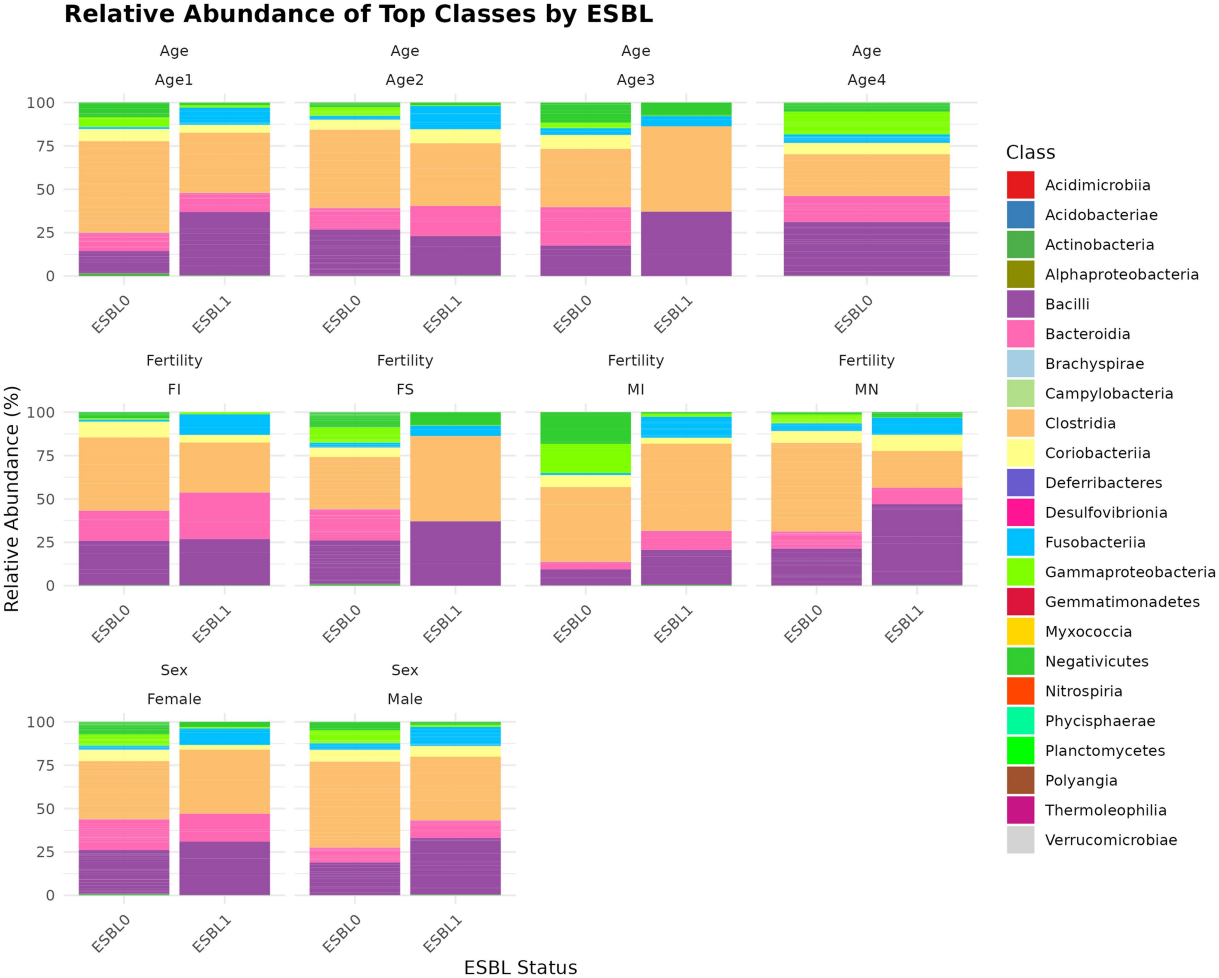
Relative abundance and composition of the gut microbiota altered at class level in ESBL-positive and ESBL-negative dogs as stratified by sex, fertility, and age. The bar plot displays taxonomic composition at the class level, comparing ESBL-positive and ESBL-negative dogs across different sex, fertility, and age groups. DESeq2 was used for statistical testing (*p* < 0.05).

A total of 22 classes (Figure 5) were identified, with the five most abundant being Clostridia (39.2%) > Bacilli (24.9%) > Bacteroidia (13.5%) > Coriobacteriia (6.27%) > Gammaproteobacteria (5.52%), which together accounted for 89.39% of the gut microbiota of the shelter dogs. Bacteroidia was 15.13% in ESBL-negative and reduced to 8.00% and ESBL-positive dogs. In contrast, Gammaproteobacteria increased from 3.93% in ESBL-negative dogs to 10.76% in ESBL-positive dogs. Gammaproteobacteria (*p =* 4.7 x E^−09^), Bacteroidia (*p =* 0.005), Deferribacteres (*p =* 0.006), Brachyspirae (*p =* 0.013), and Fusobacteria (*p =* 0.083) abundance significantly changed in dogs colonized by ESBL bacteria than the non-colonized dogs. At the class level, gut colonization by ESBL bacteria led to a significant increase in Bacilli levels in all dogs, regardless of fertility status, sex, or age except in intact females and those aged 4 to 6 months, whereas reduced Gammaproteobacteria levels in all dogs irrespective of fertility status, sex, and age (Figure 5).

#### Family

**Figure 6:**
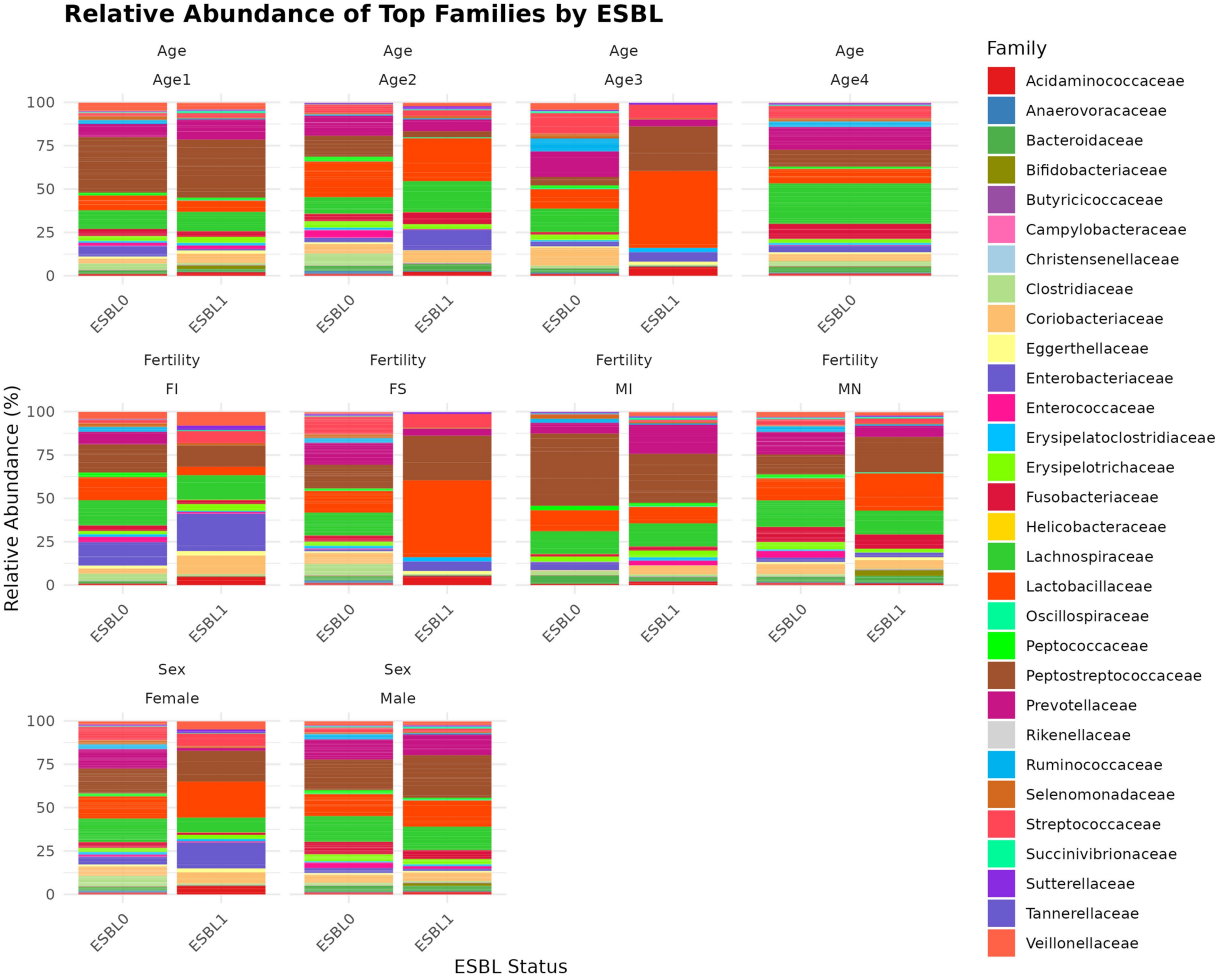
Relative abundance and composition of the gut microbiota altered at family level in ESBL-positive and ESBL-negative dogs as stratified by sex, fertility, and age. Bar plot showing the relative abundance of bacterial families in ESBL-positive and ESBL-negative dogs. Statistical significance was assessed using DESeq2 with *p* < 0.05.

A total of 119 families (Figure 6) were identified. We used two analysis methods to identify the shifted gut microbiota family due to gut colonization by ESBL bacteria, namely % abundance method that compares prevalence/proportion and DESeq2 (Log_2_ fold change) method that compares the actual counts of bacteria genera for significant differences. Accordingly, the five most prevalent families in % abundance included *Peptostreptococcaceae* (17.4%) > *Lactobacillaceae* (13.6%) > *Lachnospiraceae* (13.6%) > *Prevotellaceae* (10.8%) >*Streptococcaceae* (5.19%), which accounted for 63.59% of all microbial families in the gut of shelter dogs. *Enterobacteriaceae* was higher in ESBL-positive dogs (9.49%) than in ESBL-negative dogs (2.48%) but Prevotellaceae was lower in ESBL-positive dogs (15.46%) than in ESBL-negative dogs (12.45%). Lachnospiraceae was 12.85% in ESBL-negative dogs and 15.91% in ESBL-positive dogs. *Enterococcaceae, Enterobacteriaceae, Xanthobacteraceae, Campylobacteraceae, Fusobacteriaceae, Ruminococcaceae, Veillonellaceae, Sutterellaceae, and Lachnospiraceae* were increased 8.95, 3.84, 2.00, 1.50, 1.38, 1.36, 1.35, 1.25, and 1.23 folds in prevalence (%), respectively, in ESBL colonized shelter dogs compared to non-ESBL colonized shelter dogs, suggesting ESBL bacteria colonization promotes the abundance of these bacteria families. Conversely, the relative abundance of *Anaerovoracaceae, Succinivibrionaceae, Prevotellaceae, Streptococcaceae, Peptococcaceae, Actinomycetaceae, Desulfovibrionaceae, Oscillospiraceae, Erysipelatoclostridiaceae, Helicobacteraceae, Selenomonadaceae, Eggerthellaceae, Clostridiaceae, Butyricicoccaceae, and Rikenellaceae* were higher by 7.45, 3.18, 2.28, 2.10, 2.05, 2.0, 2.0, 1.84, 1.81, 1.8, 1.78, 1.68, 1.51, 1.5, and 1.33 folds in prevalence (%) in non-ESBL colonized shelter dogs suggesting ESBL bacteria colonization inhibits (negatively influences) the abundance of these bacteria families in shelter dogs. Based on Log_2_ fold change method by DESeq2 analysis, *Lachnospiraceae*, *Enterobacteriaceae*, and *Bifidobacteriaceae* were significantly (*p* < 0.05) increased, but *Christensenellaceae* and *Deferribacteraceae* were significantly (*p* < 0.05) reduced in ESBL colonized shelter dogs compared to non-ESBL colonized shelter dogs.

At the family level, ESBL bacteria colonization of gut significantly increased *Lactobacillaceae* levels in spayed females and dogs aged 7 to 12 months and raised *Peptostreptococcaceae* levels in spayed females. However, *Peptostreptococcaceae* levels in ESBL bacteria colonized dogs were significantly reduced in intact males and dogs aged 4 to 6 months but increased again in the 7 to 12-month age group (Figure 6).

#### Genus

**Figure 7:**
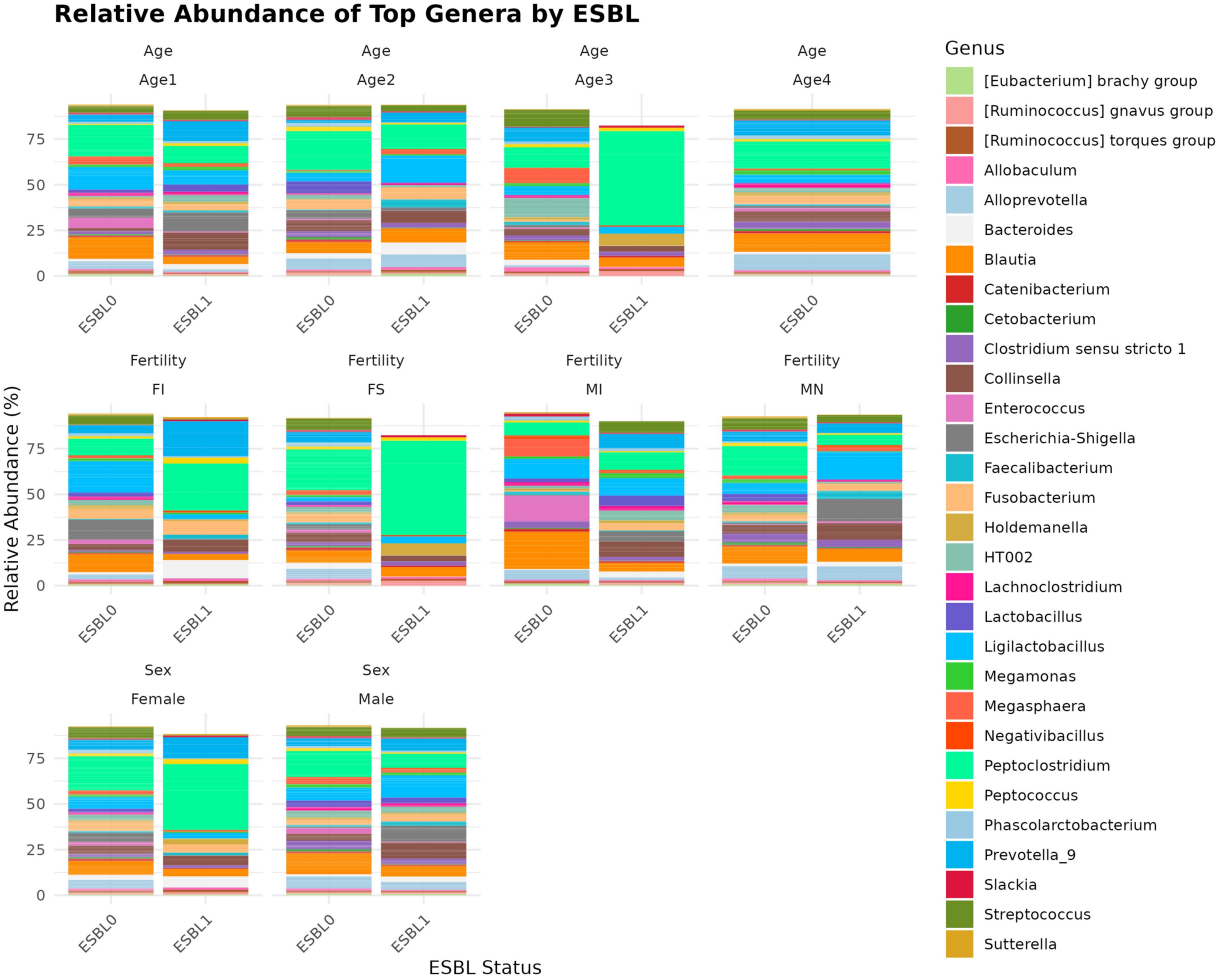
Relative abundance and composition of the gut microbiota altered at genus level in ESBL-positive and ESBL-negative dogs as stratified by sex, fertility, and age. Bar plot shows the relative abundance of bacterial genus in ESBL-positive and ESBL-negative dogs. Statistical significance was assessed using DESeq2 with *p* < 0.05.

A total of 285 genera were identified. We used two analysis methods to identify the shifted genera among gut microbiota during gut colonization by ESBL bacteria, namely % abundance method that compares prevalence/proportion and DESeq2 (Log_2_ fold change) method that compares the actual counts of bacteria genera for significant differences. Of 285 genera, five most prevalent genera based on % abundance method included *Peptoclostridium* (16.52%) > *Blautia* (8.1%) > *Ligilactobacillus* (7.36%) > *Prevotella*_9 (5.67%) > *Streptococcus* (5.17%), accounting for 42.82% of the total microbial genera. Among ESBL-positive samples, *Escherichia-Shigella* (9.48%) and *Enterococcus* (5.91%) were significantly higher compared to ESBL-negative samples (2.47 and 0.66%, respectively). In contrast, *Alloprevotella* (1.64% in ESBL-positive vs. 5.84% in ESBL-negative), *Streptococcus* (2.61% vs. 5.92%), and *Prevotella*_9 (3.71% vs. 6.24%) showed a marked decrease in ESBL-positive samples (Figure 7). At the genus level, ESBL colonization increased *Blautia* and *Ligilactobacillus* levels while reducing *Peptoclostridium* and *Lactobacillus* levels. Comparative analysis of the 285 bacteria genera in gut microbiota for abundance by DESeq2 using log_2_ fold change method between ESBL colonized dogs in comparison to ESBL non-colonized dogs showed that *Dialister*, *Lactococcus*, *Aquamonas,* and *Escherichia-Shigella* were increased significantly (*p* < 0.05) in the range of 2 to 24-fold in abundance, while *Sarcina*, *Paeniclostridium*, *Solobacterium*, *Succinivibrio*, *Christensenellaceae* R-7 group, *Brachyspira*, *Mucispirillum*, and *Slackia* were reduced significantly (*p* < 0.05) by −0.9 to −8.9 fold, respectively, indicating these genera were associated with alteration of gut microbiota during ESBL bacteria colonization.

### Beta diversity

**Figure 8:**
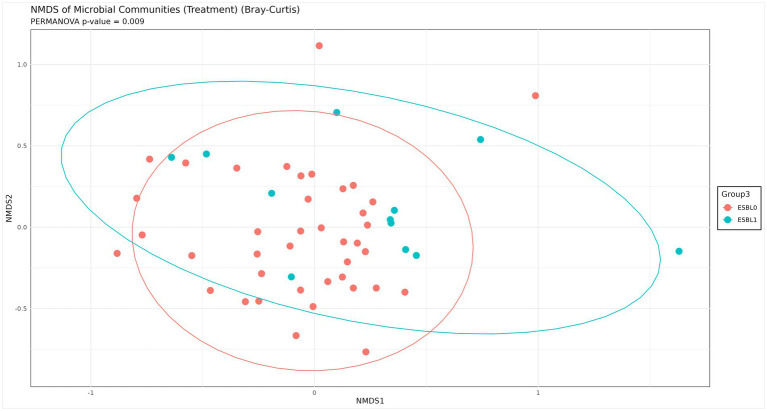
Beta-analysis by NMDS Bray-Curtis distance for distinguishing dissimilarities in microbial community composition between ESBL-bacteria colonized and non-colonized dogs. The NMDS plot visualizes differences in microbial community structure between ESBL-positive (*n* = 12) and ESBL-negative (*n* = 40) dogs, based on Bray–Curtis dissimilarity. Statistical significance was determined using PERMANOVA (*p < 0.05*).

Beta diversity analysis using Non-metric Multidimensional Scaling (NMDS) was performed using Bray-Curtis distance to distinguish dissimilarities in microbial community composition between ESBL-producing bacteria colonized dogs and non-colonized dogs. The NMDS plot revealed overlapping clusters of gut microbiota for ESBL-negative and ESBL-positive dogs, suggesting a lack of unique (distinct) microbial community patterns between the two dog groups. Despite the overlap, certain differences were noted, indicating some variations that may not be large enough to clearly separate the groups (Figure 8). The observed minor differences may reflect specific microbial shift or functional changes as hinted in other analysis in this paper.

### Gut microbiome functional and pathways changed by ESBL colonization

**Figure 9:**
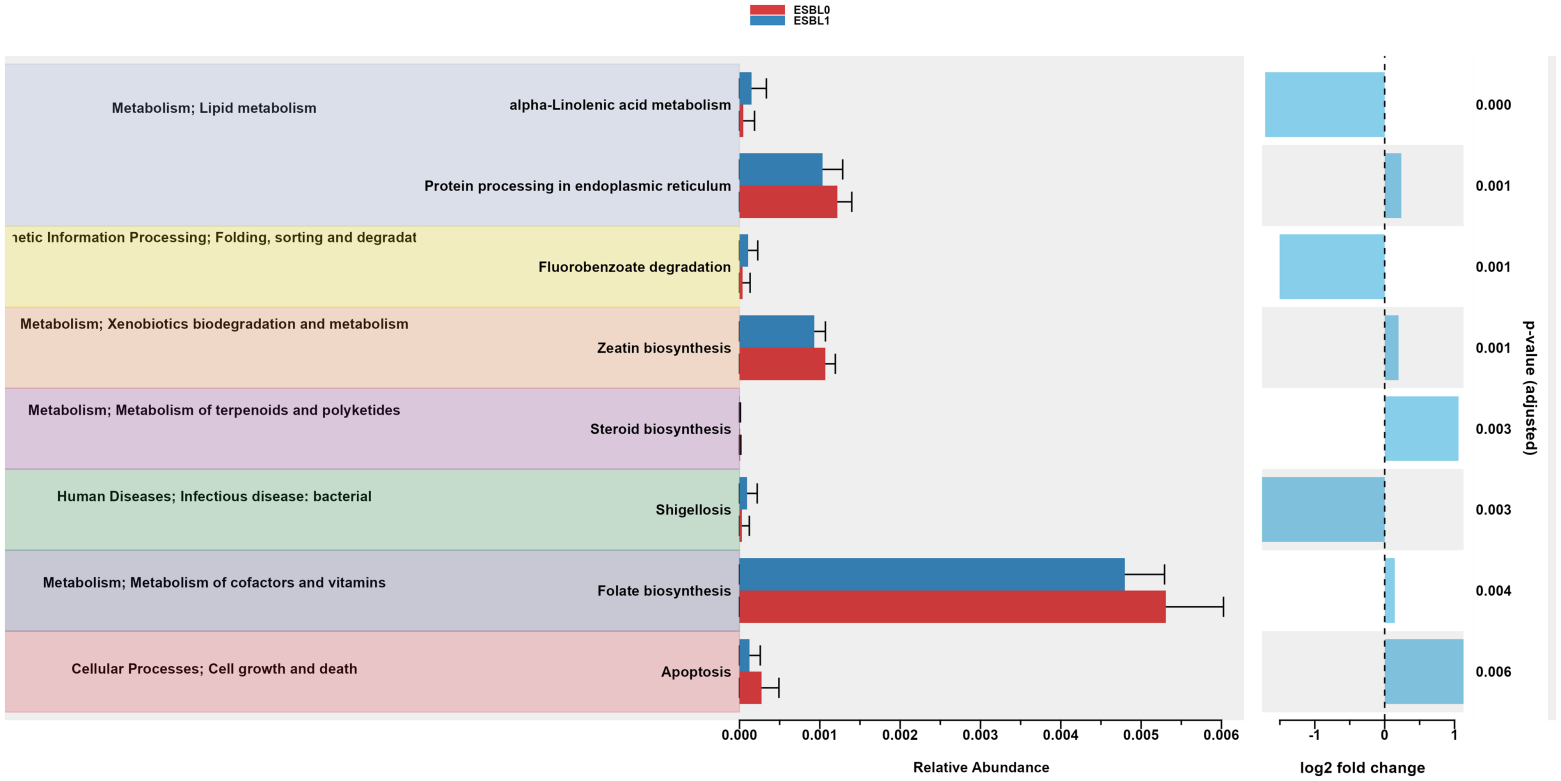
Gut microbiome functional pathways in KEGG database. PICRUSt2 analysis identified significantly altered pathways in ESBL-positive vs. ESBL-negative shelter dogs. Statistical testing was performed using Wilcoxon rank-sum test.

**Figure 10:**
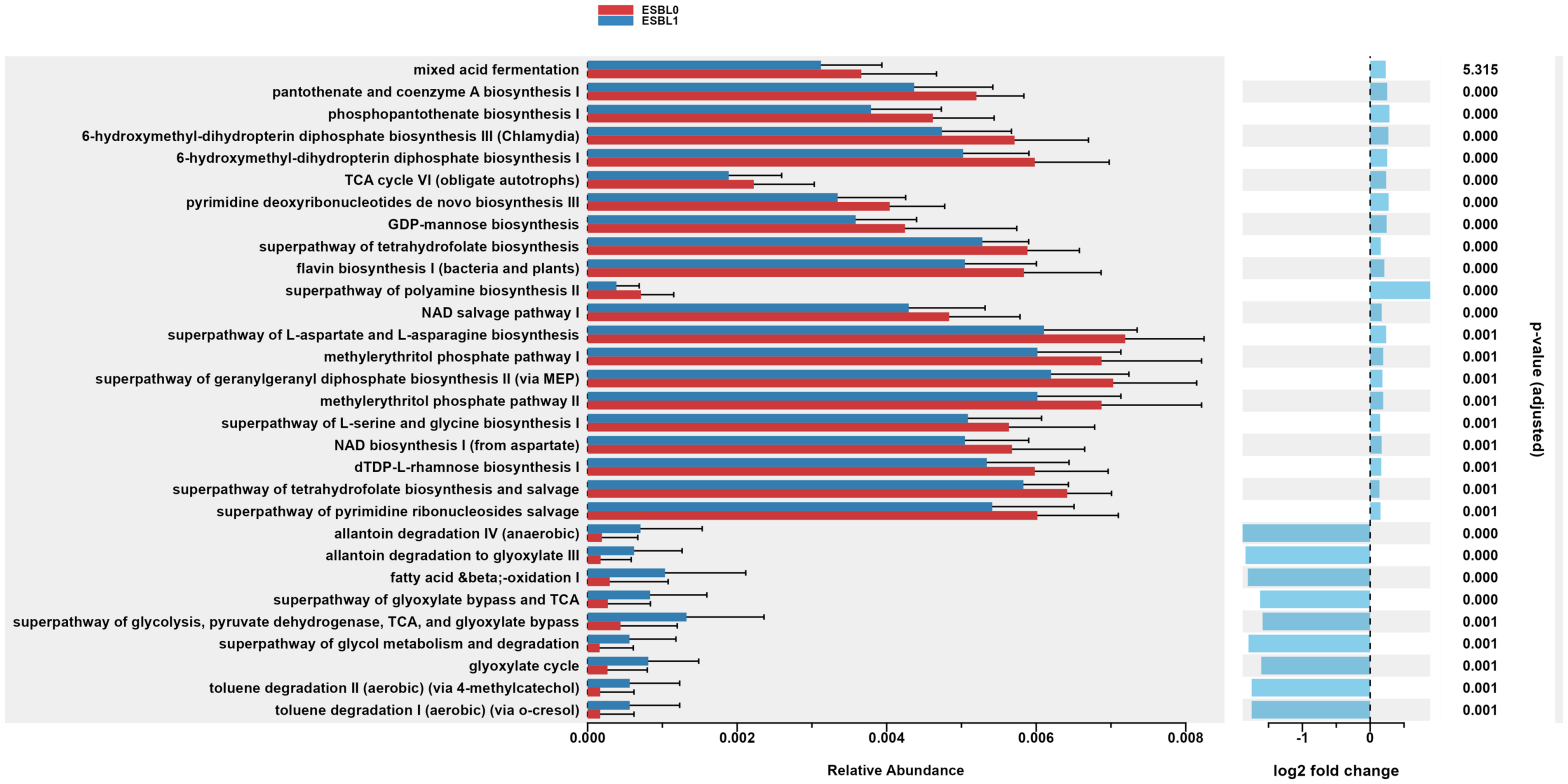
Gut microbiome functional pathways in MetaCyc database. Differentially abundant metabolic pathways identified via PICRUSt2 in ESBL-positive vs. ESBL-negative dogs. Statistical significance determined by Wilcoxon rank-sum test.

Gut microbiome functions and pathways significantly (*p* < 0.05) altered and reprogrammed by ESBL bacteria as shown by functional analysis of gut microbiome using PICRSt2, referencing KEGG (Figure 9) and MetaCyc databases (Figure 10). Most functions and pathways were reduced in ESBL-positive dogs compared ESBL-negative dogs. However, alpha-linolenic acid metabolism, fluorobenzoate degradation, and shigellosis pathways were exceptionally increased (*p =* 0.001) in dogs colonized by ESBL bacteria, referencing KEGG database (Figure 9).

Furthermore, gut colonization by ESBL-producing bacteria significantly (*p =* 0.001) upregulated the metabolic functions of allantoin degradation, toluene degradation, glycol degradation, fatty acid and beta oxidation, and glyoxylate metabolism bypass pathways compared to dogs non-colonized by ESBL bacteria, referencing MetaCyc database (Figure 10).

The increase in certain pathways may indicate the mechanisms of how ESBL-producing bacteria colonize, survive, and thrive in the gut, potentially by manipulating the microbiome to their benefit. Several pathways were reduced in ESBL-positive dogs, suggesting a downregulation of beneficial or baseline microbial functions, impairing overall gut health and microbial resilience. ESBL-negative dogs showed significantly (*p =* 0.000) higher ribosomal activity and protein processing in the endoplasmic reticulum, suggesting enhanced protein synthesis capabilities, healthier, or more robust microbial functions in non-colonized dogs.

## Discussion

Investigation is scarce or none on the effects of intestinal colonization of ESBL bacteria on gut microbiome taxa abundance, diversity, and functions in shelter dogs; hence, this study contributes to fill this gap.

In this study, we identified 6,557 OTUs in the feces of shelter dogs that are comprised of 15 phyla, 22 class, 119 families, and 285 genera indicating dogs harbor diverse microbiota in the gut. Other studies have reported that dogs harbor about 10^12^–10^14^ microbes in number in their gut microbiota (Suchodolski, 2011; Garrigues et al., 2022), comprising of 1,000 species, and encoding about 3 million genes that play many roles for the host (Rowland et al., 2018; Chen et al., 2024) such as providing enzymes for metabolic and biosynthesis pathways (Jia et al., 2022).

In our study, we observed that Firmicutes (69.3%), Bacteroidota (13.5%), Actinobacteroita (6.77%), Proteobacteria (5.54%), and Fusobacteriota (4.75%) were the top five phyla (accounting for 99.86%) of the gut microbial community in shelter dogs. Firmicutes and Bacteroidata have been consistently ranked first and second, respectively, in gut microbiota by multiple studies, but the rank order of the remaining phyla varied among different studies and vertebrate species (Binda et al., 2018; Moon et al., 2018; Rinninella et al., 2019), including dogs (Schmitz and Suchodolski, 2016; Pilla and Suchodolski, 2020; Garrigues et al., 2022; Hernandez et al., 2022). Based on literature, Firmicutes in the animal gut is composed of many bacterial families (*Clostridiaceae*, *Lachnospriaceae*, *Ruminococcoceae*, *Lactobacteriaceae*, *Enterococcoaceae*, *Staphylococcaceae*); Bacteriodoita phylum includes *Sphingobacteriaceae*, *Bacteroidaceae*, *Tannerellaceae*, *Rickenerellaceae*, and *Provotellaceae*; Actinobacteroita phylum includes *Bifidobacteriaceae*, *Coriobacteriaceae*, and *Corynebacteriaceae* (Schmitz and Suchodolski, 2016; Rinninella et al., 2019; Pilla and Suchodolski, 2020; Garrigues et al., 2022); hence, we also detected these bacteria families in gut microbiota of shelter dogs in our study.

In this study, the abundance of some bacterial phylum in the gut were shifted in dogs colonized with ESBL bacteria compared to non-colonized dogs, where dog demographic factors (age, sex, and fertility status) further modified the abundance levels of gut microbiota, which agrees with study on dogs in Netherlands (Stege et al., 2023). The most significantly shifted microbiota in abundance includes the following bacterial phyla (Proteobacteria, Deferribacterota, Bacteroidota, Fusobacteriota, and Spirochaetota). Shift in abundance was also noticed in the following bacterial class (Gammaproteobacteria, Bacteroidia, Deferribacteres, Brachyspirae, and Fusobacteria) in ESBL colonized shelter dogs compared to non-ESBL colonized shelter dogs. Based on family, the levels of *Enterococcaceae*, *Enterobacteriaceae*, and *Xanthobacteraceae* families have increased, but the decline of *Anaerovoracaceae, Succinivibrionaceae, Prevotellaceae, Streptococcaceae, Peptococcaceae, Actinomycetaceae, Desulfovibrionaceae* in ESBL colonized shelter dogs have been noticed in ESBL colonized shelter dogs compared to non-ESBL colonized shelter dogs, indicating ESBL bacteria manipulate gut microbiota bacterial families for colonization and survival in shelter dogs. Studies have shown that Bacteroidota (Shin et al., 2024; Tufail and Schmitz, 2024) and Actinobacteriota (including its Bifidobacteria) (Binda et al., 2018) are beneficial for gut microbiota stability, indicating their decline marks disrupted gut microbiota homeostasis. In this study, gut colonization by ESBL bacteria resulted in a decline of the healthy gut microbial indicators (e.g., Bacteriodata) (Shin et al., 2024; Tufail and Schmitz, 2024) and increase in gut microbial dysbiosis indicator (e.g., Proteobacteria) (Shin et al., 2015). The shelter dogs with lowered Bacteroidata levels may be at risk to ill health since Bacteriodata maintain the integrity of normal/healthy digestive tract, breakdown complex polysaccharides in nutrient, have a probiotic potential, and protect the gut from pathogen infections by synthesizing various metabolites such as fatty acids and secondary bile acids although they potentially become pathogenic outside of the gut and in damaged gut (Shin et al., 2024; Tufail and Schmitz, 2024). Proteobacteria levels in gut is normally low (Shin et al., 2015), but we noticed increased levels of Proteobacteria and *Enterobacteriaceae* in dogs colonized by ESBL bacteria in this study suggesting gut microbiota of dogs colonized by ESBL bacteria were destabilized. A study has reported that humans and pet dogs show similarity in gut microbiota disturbances, characterized by a reduction in microbial diversity, a reduction in Firmicutes and an increase in Proteobacteria; a reduction in short-chain fatty acids (SCFAs); an increase in primary bile acids (BAs); and a reduction in secondary BAs (Hernandez et al., 2022). Since *Enterobacteriaceae* family harbors diarrheagenic *Escherichia coli*, *Campylobacter jejuni*, *Klebsiella pneumoniae*, *Salmonella typhimurium*, and *Yersenia enterocolitica*, the increased *Enterobacteriaceae* levels in ESBL bacteria colonized dogs can be an indicator or risk for gut dysbiosis, aging, AMR dissemination and zoonotic transmission to adopters (Moon et al., 2018; Rinninella et al., 2019; Baldelli et al., 2021; Xu et al., 2023). Given that the causative agents of gut dysbiosis are prevalent in supply chain of shelter dogs as reviewed elsewhere, the gut dysbiosis inducer agents could have altered the gut microbiota promoting ESBL bacteria colonization of the gut for alteration of gut microbiota of shelter dogs or ESBL bacteria might have acquired weaponry that alter gut microbiota (Stege et al., 2023). Since many confounding factors from host genetic and environmental factors involve in gut microbiome taxa and functional shift and disruption (Spor et al., 2011), this study did not determine the underlining causality (temporality) as to whether ESBL bacteria colonization or unknown factor (e.g., stress or antimicrobial treatment) caused the observed gut microbiota disruption in dogs colonized by ESBL bacteria; hence, a controlled trial is needed. Whatever the underlining cause, restoring the homeostasis of gut microbiota is important. Stool transplant can serve as a “live medicine” to prevent AMR bacteria colonization/invasion (Ubeda et al., 2013; Xu et al., 2015; Ooijevaar et al., 2019; Biazzo and Deidda, 2022) as well to repair the gut ecosystem and eradicate harmful pathogens from the gut by *Bifidobacteria*, *Bacillus subtilis*, *Lactobacillus*, and other anaerobic microbiota in the stool transplant (McKenney and Pamer, 2015; Pamer, 2016; Gagliardi et al., 2018; Ubeda et al., 2013; Singh et al., 2014; Pamer, 2016; Juhász et al., 2021; Wuethrich et al., 2021). To improve the gut health and stabilize gut microbiota, some researchers have prescribed different bacteria (probiotics) that benefits the dogs through producing antimicrobial metabolites, inhibiting colonization by harmful pathogens, inhibiting toxin production and destroying toxins produced by pathogens, and increasing abundance of mucosal antibodies (IgA) (Schmitz and Suchodolski, 2016). Of many probiotic bacteria, the most widely used ones include eight *Lactobacilli* (*L. acidophilus, L. casei, L. plantarum, L. paracasei, L. lactis, L. rhamnosus, L. salivarius, L. delbrueckii* ssp. bulgaricus), five *Bifidobacteria* (*B. animalis*, *B. breve*, *B. bifidum*, *B. longum*, *B. infantis*), two Bacillus (*B. subtilis*, *B. coagulans*), two *Enterococcus faecium* strains, and *Streptococcus thermophilus* (Schmitz and Suchodolski, 2016).

We noticed that the abundance levels of *Enterobacteriaceae*, *Peptostreptococcaceae*, *Lactobacillaceae*, *Lachnospiraceae*, *Prevotellaceae*, and *Peptostreptococcaceae* bacterial family in ESBL bacteria colonized and noncolonized shelter dogs varied due to the modifying effect of age, sex, and fertility status (intact vs. spayed/neutered). Information is scarce concerning the effect of interaction of ESBL colonization and dog co-variates on gut microbiota to contrast. However, the abundance, composition, and spatial distribution of gut microbiota vary by age (Saraswati and Sitaraman, 2014; Walker et al., 2015; Biragyn and Ferrucci, 2018; de la Cuesta-Zuluaga et al., 2019; Mizukami et al., 2019; Yoon and Kim, 2021; Ma et al., 2023), sex (Jašarević et al., 2017; de la Cuesta-Zuluaga et al., 2019; Jaggar et al., 2020; Scarsella et al., 2020; Koliada et al., 2021; Yoon and Kim, 2021; Kim, 2022; Ma et al., 2023), and fertility status (Jašarević et al., 2017; Yuan et al., 2020; Yang K. et al., 2023). Sex of the host has effect on gut microbiota and reciprocally microbiota also influences the sex hormones of the host (Jaggar et al., 2020). *Lachnospiraceae*, *Prevotellaceae*, and *Bifidobacterium* are known for producing beneficial metabolites for the gut/animals such as short chain fatty acids (propionate, butyrate, and acetate) (Vacca et al., 2020; Montgomery et al., 2024), polysaccharide breakdown (Precup and Vodnar, 2019).

At genus level, colonization with ESBL bacteria is linked to notable shift in gut microbiota composition. Accordingly, we noticed a significant increase in actual number of *Dialister*, *Lactococcus*, *Aquamonas*, and *Escherichia-Shigella* and a significant decline of *Sarcina*, *Paeniclostridium*, *Solobacterium*, *Succinivibrio*, *Christensenellaceae* R-7 group, *Brachyspira*, *Mucispirillum*, and *Slackia* in gut microbiota in ESBL bacteria colonized dogs. Previous studies have indicated that these genera are among gut microbiota that are mostly subjected or linked to altered gut microbiota by multiple factors such as diet (Hooda et al., 2013; Do et al., 2021; Pilla and Suchodolski, 2021), where some of these genera are both beneficial and harmful depending on the context in literature as described below. *Dialister* belongs to *Veillonellaceae* family in Firmicutes Phylum (Afouda et al., 2020). The increase *Dialister* abundance has association with diseases such as obesity (Pinart et al., 2022) and spondyloarthritis (Tito et al., 2017). *Aquamonas* belongs to betaproteobacterium that live in aquatic environment (Bereschenko et al., 2008), but infrequently detected in gut microbiota of animals with unknown role in the gut (Lee and Hwang, 2025). *Sarcina* belongs to the *Clostridiacee family* and produces gas and short-chain fatty acids (SCFAs) in the gut by fermenting fibers/carbohydrate diet, but it sometimes become pathogenic causing bloat (emphysematous), gastric dilation, and delayed gastric emptying in humans and animals (Al Rasheed and Senseng, 2016; Tartaglia et al., 2022; Makovska et al., 2023). *Paeniclostridium* is a pathogenic *Clostridium* in gut microbiota that causes severe gut edema and hemorrhage, extreme leukemoid reaction, and lack of an innate immune response in dogs (Capewell et al., 2020; French et al., 2022). *Solobacterium* belongs to *Erysipelotrichidae* family in Firmicutes phylum that produces bad breath due to its link with bad breath smell (H2S and CH_3_SH production) and periodontal disease in humans (Barrak et al., 2020; Bachtiar et al., 2022). Its increase in the gut can be used as markers of colorectal cancer in humans (Kapsetaki et al., 2022), but *Solobacterium* is not well studied in dogs. *Succinivibrio* and *Escherichia-Shigella* are Protobacteria in phylum Firmicutes. *Succinivibrio* are abundant in the gut of dogs (Handl et al., 2011; Alessandri et al., 2020), their abundance increases in fiber diets fed dogs than in meat fed dogs (Moon et al., 2018). *Succinivibrio* ferments carbohydrates to produce succinate, digests fibers, and stabilizes gut ecosystem by reducing methane production by competing with methanogenic bacteria in the gut, where their increase in rumen also increases milk yield in cows (Hailemariam et al., 2020) but their increase in human gut maybe harmful as it links with human inflammatory diseases (Tecer et al., 2020). *Christensenellaceae* R-7 Group is in phylum Firmicutes that is considered a beneficial bacterium due to its association with low body mass index (BMI) (i.e., lean body), body fat change, and healthy gut function (Waters and Ley, 2019; Jian et al., 2022). *Mucispirillum* belongs to Deferribacterota phylum that colonizes the mucus layer of the intestinal epithelium, providing protection to the gut against harmful pathogen colonization (Herp et al., 2019), but they can be pathogenic if they acquire of novel genes or their number increased in the gut (Loy et al., 2017). *Slackia* belongs to *Coriobacteriaceae* family in Actinobacteria phylum (Clavel et al., 2014). *Slackia* is beneficial in bile acid metabolism and production of bioactive metabolites such as isoflavonoids, e.g., equol (Mayo et al., 2019; Gao et al., 2020), but it can be pathogenic (Kumaresan et al., 2024). In general, the dual nature of microbiota in the gut (being beneficial in some contexts and harmful in others) highlights the complex interplay between ESBL colonization, gut microbiota balance, and host health.

The functional pathways of the gut microbiota varied between ESBL-negative and ESBL-positive dogs, irrespective of dog age, sex, and fertility status in this study. Most metabolic and biosynthetic pathways in the gut microbiome were downregulated in dogs colonized by ESBL bacteria compared to ESBL-negative dogs. However, pathways such as alpha-linolenic acid metabolism and shigellosis, fluorobenzoate degradation, allantoin degradation, toluene degradation, glycol degradation, fatty acid and beta oxidation, and glyoxylate metabolism bypass pathways were increased in dogs colonized by ESBL bacteria. The shift in gut microbiota metabolic and biosynthesis pathways can be explained by the up and down regulated abundance of different bacterial phyla, class, family, and genera described in this study. This study did not determine the responsible gut bacteria for the shift in functional since it is challenging due to the presence of 10^12^–10^14^ microbes in number in the gut (Suchodolski, 2011; Garrigues et al., 2022) that belong to 1,000 microbe species and collectively carry over 3 million genes encoding diverse enzymes, metabolites, metabolic and biosynthesis pathways (Krishnan et al., 2015; Rowland et al., 2018; Jia et al., 2022; Chen et al., 2024). The second limitation of our study is that it was an observational study. A randomization-controlled trial (RCT) is a gold standard in clinical research, offering greater control over patient allocation, confounding variables, and bias, thereby enabling stronger causal inference between gut microbiome alteration and ESBL bacteria colonization. However, conducting RCT by exposing shelter dogs experimentally to ESBL bacteria would be considered unethical and shelter managers may not be willing to do it. Thus, our observational study, despite its limitation, remains the most ethical, acceptable, and feasible approach given our access to shelter dogs. The third limitation is that we relied on 16S gene sequencing for gut microbiota and functional microbiome profiling. While this method provides valuable insights, whole shotgun metagenomic sequencing is a more powerful, allowing for the identification of low-abundant taxa and strains while providing sequences of all genes on the genomic DNA of all organisms in the gut. However, we were unable to use whole shotgun metagenomic sequencing in our study due to budget issues.

In conclusion, shifts in the abundance level of gut *Enterobacteriaceae*, *Peptostreptococcaceae*, *Lactobacillaceae*, *Lachnospiraceae*, *Prevotellaceae*, *Peptostreptococcaceae* and as well as multiple bacteria genera have been observed in ESBL bacteria colonized shelter dogs. These shifts in taxa may reflect the gut microbiome’s response to ESBL bacteria colonization and reducing of the abundance of beneficial commensals. ESBL-producing bacteria apparently colonize the gut by disrupting/downregulating numerous key microbial metabolic and biosynthesis pathways (whereas by upgrading a few or some of them). These functional shifts can potentially reshape and reprogram the gut microbial ecosystem, favoring the survival of “bad” microbiota (i.e., ESBL bacteria) while simultaneously reducing the gut’s overall “good” microbial functional capacity such as nutrient synthesis, immune function, pathogen defense, and implication on dog’s health.

## Data Availability

The original contributions presented in the study are publicly available. This data can be found here: https://www.ncbi.nlm.nih.gov/, accession number: PRJNA1234399.

## Footnotes

^1^ eco-pet-demographic-report-22-low-res.pdf (avma.org)

^2^ https://www.bioinformatics.babraham.ac.uk/projects/fastqc/

^3^ https://www.arb-silva.de/

^4^ https://www.kegg.jp

^5^ MetaCyc.org

^6^ Index of /src/contrib/Archive/vegan
